# Clinical and Histopathological Features of Patients with Systemic Sclerosis Undergoing Endomyocardial Biopsy

**DOI:** 10.1371/journal.pone.0126707

**Published:** 2015-05-12

**Authors:** Karin A. L. Mueller, Iris I. Mueller, David Eppler, Christine S. Zuern, Peter Seizer, Ulrich Kramer, Ina Koetter, Martin Roecken, Reinhard Kandolf, Meinrad Gawaz, Tobias Geisler, Joerg C. Henes, Karin Klingel

**Affiliations:** 1 Medizinische Klinik III, Kardiologie und Kreislauferkrankungen, Eberhard Karls Universitaetsklinikum Tuebingen, Tuebingen, Germany; 2 Institut für Radiologie, Eberhard Karls Universitaetsklinikum Tuebingen, Tuebingen, Germany; 3 Centre for Interdisciplinary Clinical Immunology, Rheumatology and Autoimmune Diseases – INDRA and Department of Internal Medicine II (Oncology, Haematology, Immunology, Rheumatology, Pulmonology), Eberhard Karls University Hospital Tuebingen, Germany; 4 Hautklinik, Eberhard Karls Universitaetsklinikum Tuebingen, Tuebingen, Germany; 5 Abteilung für Molekulare Pathologie, Eberhard Karls Universitaetsklinikum Tuebingen, Tuebingen, Germany; University of Texas Health Science Center at Houston, UNITED STATES

## Abstract

**Background:**

Cardiac involvement in systemic sclerosis (SSc) is associated with a variable phenotype including heart failure, arrhythmias and pulmonary hypertension. The aim of the present study was to evaluate clinical characteristics, histopathological findings and outcome of patients with SSc and a clinical phenotype suggesting cardiac involvement.

**Methods and Results:**

25 patients with SSc and clinical signs of cardiac involvement were included between June 2007 and December 2010. They underwent routine clinical work-up including laboratory testing, echocardiography, left and right heart catheterization, holter recordings and endomyocardial biopsy. Primary endpoint (EP) was defined as the combination of cardiovascular death, arrhythmic endpoints (defined as appropriate discharge of implantable cardioverter defibrillator (ICD)) or rehospitalization due to heart failure. The majority of patients presented with slightly impaired left ventricular function (mean LVEF 54.1±9.0%, determined by echocardiography). Endomyocardial biopsies detected cardiac fibrosis in all patients with a variable area percentage of 8% to 32%. Cardiac inflammation was diagnosed as follows: No inflammation in 3.8%, isolated inflammatory cells in 38.5%, a few foci of inflammation in 30.8%, several foci of inflammation in 15.4%, and pronounced inflammation in 7.7% of patients. During follow up (FU) (22.5 months), seven (28%) patients reached the primary EP. Patients with subsequent events showed a higher degree of fibrosis and inflammation in the myocardium by trend. While patients with an inflammation grade 0 or 1 showed an event rate of 18.2%, the subgroup of patients with an inflammation grade 2 presented with an event rate of 25% versus an event rate of 50% in the subgroup of patients with an inflammation grade 3 and 4, respectively (p=0.193). Furthermore, the subgroup of patients with fibrosis grade 1 showed an event rate of 11%, patients with fibrosis grade 2 and 3 presented with an event rate of 33% and 42% respectively (p = 0.160).

**Conclusions:**

Patients with SSc and clinical signs of cardiac involvement presented with mildly impaired LVEF. Prognosis was poor with an event rate of 28% within 22.5 months FU and was associated with the degree of cardiac inflammation and fibrosis.

## Introduction

Systemic sclerosis (SSc) is a rare chronic disease of unknown cause characterized by diffuse fibrosis, degenerative changes, and vascular abnormalities in the skin, joints, and internal organs including the heart. Primary cardiac involvement in progressive SSc remains clinically silent for a long time period, but is associated with a very poor prognosis, when first symptoms become apparent [1–3]. While prognosis of SSc improved over the last decades, cardiac mortality did not decrease and remains one of the major causes of death in SSc with up to 70% [4–8]. The pathological involvement of myocardium and risk assessment in patients with SSc has been poorly evaluated so far.

Patients with limited (lc) or diffuse cutaneous (dc) SSc are characterized by clinically evident, inflammatory, and fibrotic processes of the skin. These mechanisms also play a major role in cardiac involvement leading to various clinical manifestations like heart failure, arrhythmias and pulmonary hypertension. Especially conductive disorders and ventricular tachycardia are described in patients with SSc implying, that these patients are at risk for cardiac events and might benefit from prevention of sudden cardiac death [9]. Patients with dc SSc are at higher risk to develop cardiac involvement than those with lc manifestations [10].

The affection of myocardium due to SSc is classified as secondary cardiomyopathy. So far, the prognosis of secondary cardiomyopathies [7–9] was associated with its underlying disease, but also with the degree of cardiac inflammation, fibrosis and subsequent dysfunction of the myocardium as well as impairment of the left ventricular function [2]. Therefore, some advocate the implantation of cardioverter defibrillators (ICDs) at an early stage of the disease, as these patients are at a high risk to develop ventricular arrhythmias and finally sudden cardiac death [9]. Hence, intensified risk stratification is needed to identify patients at risk to improve their outcome and prognosis. Recent cases of sudden cardiac death led to a more intensive screening for occult cardiac involvement, especially in patients planned for autologous stem cell transplantation (aSCT) [11].

Therefore, the aim of the present study was to evaluate clinical characteristics, histopathological findings of endomyocardial biopsies, and its prognostic value in patients with SSc and suspected cardiac involvement to improve cardiac risk assessment.

## Materials and Methods

### Study design, patient collective and assessment of clinical risk factors

A retrospective analysis was performed including 25 patients with SSc, who underwent endomyocardial biopsy as part of the routine clinical evaluation for suspected cardiac involvement between June 2007 and December 2010. All patients were admitted or transferred to the University Hospital Tuebingen due to clinical signs of suspected cardiac involvement of SSc [12,13], with either exertion dyspnoe NYHA classification ≥ 2, and/or elevated troponin I (TnI) levels > 0.03μg/l and/or elevated B-type natriuretic peptide (BNP) levels >100ng/l, and/or cardiac arrhythmias. Indications for endomyocardial biopsy were based on these clinical criteria mentioned above (persistently elevated cardiac markers or documented ventricular arrhythmias) or one of the following clinical indications: new-onset heart failure of 2-week duration with dilation of the left ventricle and hemodynamic compromise, new-onset heart failure of up to 3-month duration with dilated left ventricle and malignant arrhythmias or failure to respond to usual care or suspected cardiac involvement with impaired global or regional systolic left or right ventricular function, enlargement of the left or right ventricle, pericardial effusion, myocardial hypertrophy, or abnormal myocardial echo patterns in transthoracic echocardiography (TTE) suggesting myocardial involvement of SSc.

All patients were diagnosed with SSc by experienced rheumatologists. They all fulfilled the ACR (American College of Rheumatology) classification criteria 2013 [14]. Patients presented with either dc or lc SSc. Disease duration was measured based on non-raynaud’s manifestations onset.

Since all patients were diagnosed with SSc, the cardiac involvement in our patient cohort is a secondary cardiomyopathy due to an underlying systemic disease. The definition of cardiomyopathies, proposed by the AHA expert consensus panel [12] and similar to that reported by the European Society of Cardiology (ESC) [13], supports our classification. In our analysis, we are focusing on patients with secondary cardiomyopathy due to the generalized disorder, but we did not include patients with indirect cardiac involvement, i.e. right heart failure caused by pulmonary arterial hypertension (PAH), or hypertensive heart disease. These indirect causes for the cardiac involvement could be ruled out in most patients. There remain confounders like arterial hypertension and elevated pulmonary arterial pressure (PAP), but a hallmark of SSc is heterogeneity due to various organ manifestations also within the cohort.

Five patients presented with arterial hypertension, which was well controlled at study entry and at the time of endomyocardial biopsy with a blood pressure (BP) <140/90mmHg in the documented BP measurements. Five patients showed a mean pulmonary arterial pressure (PAmean) > 25mmHg in right heart catheterization. The elevated PAmean is expression of the underlying pulmonary fibrosis detected in 19 (76%) patients by high resolution computed tomography (hrCT) and not due to left sided heart failure.

Hypertensive heart disease or cor pulmonale due to pulmonary fibrosis can lead to fibrotic remodelling within their myocardium and are potential confounders. But in our endomyocardial biopsies, we did not only analyse the degree of fibrosis but also the degree of inflammation suggesting an inflammatory response within the myocardium, which is so far not described in hypertensive cardiomyopathy or cor pulmonale. Moreover, typical findings of hypertensive or hypertrophic cardiomyopathy were not observed in our SSc patients [15] suggesting another underlying cause for the cardiac involvement than SSc [16].

All patients underwent TTE, left and right heart catheterization, pulmonary function tests and 24 hours holter recordings (n = 18) for further diagnostic workup at study entry.

Patients´ history, physical examination, laboratory testings and autoantibody status (ANA, PmScl, Anti-Jo and SSA or SSB, anti-Scl70- and anti-centromere-antibodies (ab) (ACA)) were collected in all subjects at study enrollment. Autoantibodies were measured qualitatively by immunofluorescence and immunodiffusion before biopsy. Clinical risk factors at study entry included age, gender, body mass index (BMI), NYHA functional class, and concomitant medication. TnI (normal value < 0.03μg/l), BNP (normal value < 100ng/l), creatine kinase (CK) (normal value < 190 U/l) and C-reactive protein (CRP) (normal value < 0.5mg/dl) were assessed as laboratory markers. Pulmonary function tests were performed at baseline in all patients to assess functional vital capacity (FVC, measured in liter and percent predicted). The modified Rodnan skin score (mRSS; range from 0 to 51) served as clinical measure for progression of skin fibrosis.

Renal involvement was diagnosed, if a glomerular filtration rat (GFR) <90ml/min/1.73 m^2^ was calculated and/or if creatinine clearance in 24h urine specimen was < 90-140ml/min.

There is no adequately clear definition of renal crisis. We diagnosed renal crisis, when typical symptoms occured. Patients usually present with poorly controlled hypertension and progressive renal impairment. The presence of hypertension is not mandatory, and there are reports of normotensive renal crisis with poor outcome in the literature [17–20]. Other clinical features are hypertensive retinopathy and encephalopathy [21], which can occur at low levels of hypertension or even at normotension, suggesting abnormal endothelial function in vessels outside the kidneys. Microangiopathic haemolytic anaemia is also a common finding. Urinalysis reveals non-nephrotic range proteinuria and haematuria. Granular casts can often be seen on microscopy. Renal failure is a typical complication, but often progresses over weeks rather than days. Approximately two-thirds of the cases with renal crisis require at least intermediate renal replacement therapy [22].

Pulmonary fibrosis was detected bei hrCT as described before [23]. Oesophageal dysfunction was diagnosed by barium-oesophagogram [24]. Furthermore, myositis was diagnosed in 11 (44%) patients by whole body magnetic resonance imaging (MRI) as described before [25]. In these patients elevated CK was found. Mean CK within these patients was 782.6 ± 601.2 U/l. CK-MB was not performed, because the clinical diagnosis was apparent regarding the patients’s symptoms. As myositis is commonly found in SSc and no further myositis associated autoantibodies (e.g. Jo1-AB) were found in these patients, we resigned to perform muscle biopsy to rule out other overlapping syndromes, especially because there were no other symptoms or clinical evidence for another underlying causes in these 11 patients. There might be correlations between myositis and cardiac involvement, but to date there are only limited data available. We did not find any typical histiopathological patterns suggesting any correlation.

Echocardiographic parameters included left ventricular ejection fraction (LVEF), left ventricular enddiastolic diameter (LVEDD), right ventricular function (RVEF), right ventricular enddiastolic diameter (RVEDD), and systolic pulmonary arterial pressure (PAPsys).

LVEF was estimated by echocardiography (iE33, Philips Medical Systems) using the modified Simpson rule with images obtained from apical 4- and 2-chamber views. RVEF was estimated using M-mode analyzing the tricuspid annular plane systolic excursion (TAPSE) by an experienced investigator, TAPSE <20 mm was defined as RV systolic dysfunction [26,27]. LVEDD and RVEDD were analyzed by 2-dimensionally guided M-mode echocardiography in all patients. PAPsys was calculated from the transtricuspid pressure gradient, as measured by continuous wave Doppler, after the addition of an estimated right atrial pressure. A commonly employed method was used by determining the variation in the size of the inferior vena cava with inspiration, i.e.: complete collapse = right atrial pressure = 5mmHg, partial collapse, right atrial pressure = 10mmHg, and no collapse, right atrial pressure = 15mmHg [28].

Significant coronary artery disease (> 50% diameter luminal stenosis of two or more coronary vessels or left main or proximal left anterior descending coronary artery stenosis > 50%) was ruled out by coronary angiography in all patients.

All patients were medically treated according to current guidelines depending on degree of heart failure symptoms and left ventricular function status [29].

No patient received hydroxychloroquine or anti-tumor necrosis factor (anti-TNF) antibodies at any point of patient’s history.

The study conformed to the principles outlined in the Declaration of Helsinki, written informed consent was obtained from all patients and the study was approved by the local ethical committee of the Eberhard Karl University Tuebingen (95/2009BO1).

### Study end points and follow-up

Patients presented in our outpatient clinic for clinical follow-up (FU) scheduled every 3 to 6 months. Patients, who missed their FU visit were contacted by telephone for an interview. None of the patients was lost to FU. Mean FU was 22.5 months.

Primary study endpoint (EP) was a combination of cardiovascular death, arrhythmic endpoints (defined as appropriate discharge of ICD) or re-hospitalization due to heart failure.

### Endomyocardial biopsy, histopathological and immunohistological analysis

Biopsy sample site was the septum of the right ventricle in all patients and at least six specimens with a diameter of 1 to 3 mm were harvested. Biopsy samples were taken with a dedicated bioptome (Biopsy Forceps, Cordis Corporation) advanced through 9 French venous sheath. Samples were immediately fixed under sterile conditions in 4% buffered formaldehyde for routine light microscopy examination regarding histology and immunohistology using hematoxylin and eosin (HE), Masson’s trichrome, Giemsa, Kongo red staining, and immunohistochemical SM-actin (smooth muscle actin) staining. 4-μm-thick paraffin-embedded tissue sections were examined by light microscopy [30]. Serial sections were obtained for the analysis. Other samples were fixed in RNAlater (Ambion Inc, Foster City, Calif) for (RT-) polymerase chain reaction PCR detection of viral genomes [10]. The quality of the biopsies was good, allowing all histological, immunohistological and molecular biological investigations in all patients according to requirements as stated by Leone et al. [31] and Basso et al. [32].

An avidin-biotin-immunoperoxidase method according to the manufacturer’s protocol (Vectastain Elite ABC Kit, Vector, Burlingame, Calif) was used for immunohistochemistry comprising the following monoclonal antibodies to identify, localize and characterize mononuclear cell infiltrates: CD3 for T-cells (Novocastra Laboratories, Newcastle on Tyne, UK), CD68 for macrophages (DAKO, Glostrup, Denmark), and HLA-DR-α (DAKO, Hamburg, Germany) to assess major histocompatibility complex (MHC) class II expression in antigen-presenting immune cells. The analysis of inflammation was done according to the World Health Organization/International Society and Federation of Cardiology Task Force on the Definition and Classification of Cardiomyopathies: Endomyocardial biopsies were considered for presence of inflammation after immunohistochemical detection of focal or diffuse mononuclear infiltrates with ≥14 per 1 mm^2^ immune cells (CD3-T-lymphocytes and/or CD68-macrophages) in the myocardium, in addition to enhanced expression of MHC class II molecules [33].

The described histological standard methods allow diagnosis of, e.g. myocarditis, dilated cardiomyopathy, and amyloidosis. In case of inflammatory heart disease, it is necessary to specify inflammatory cell subtypes by immunohistochemistry to differentiate e.g. virus-induced myocarditis from giant cell myocarditis or eosinophilic myocarditis according to the guidelines of the Association for European Cardiovascular Pathology [31]. This means, that basic histologic and immunohistological stainings are used in complement to receive the specific diagnosis of the underlying heart disease.

Furthermore, we refined the degree of inflammation according to a modified scheme as described [34–36]:

grade 0 = no inflammation

grade 1 = single inflammatory cells (T-lymphocytes and macrophages ≥14/mm^2^) grade 2 = a few foci of inflammation

grade 3 = several foci of inflammation

grade 4 = pronounced inflammation

**Fig 1:**
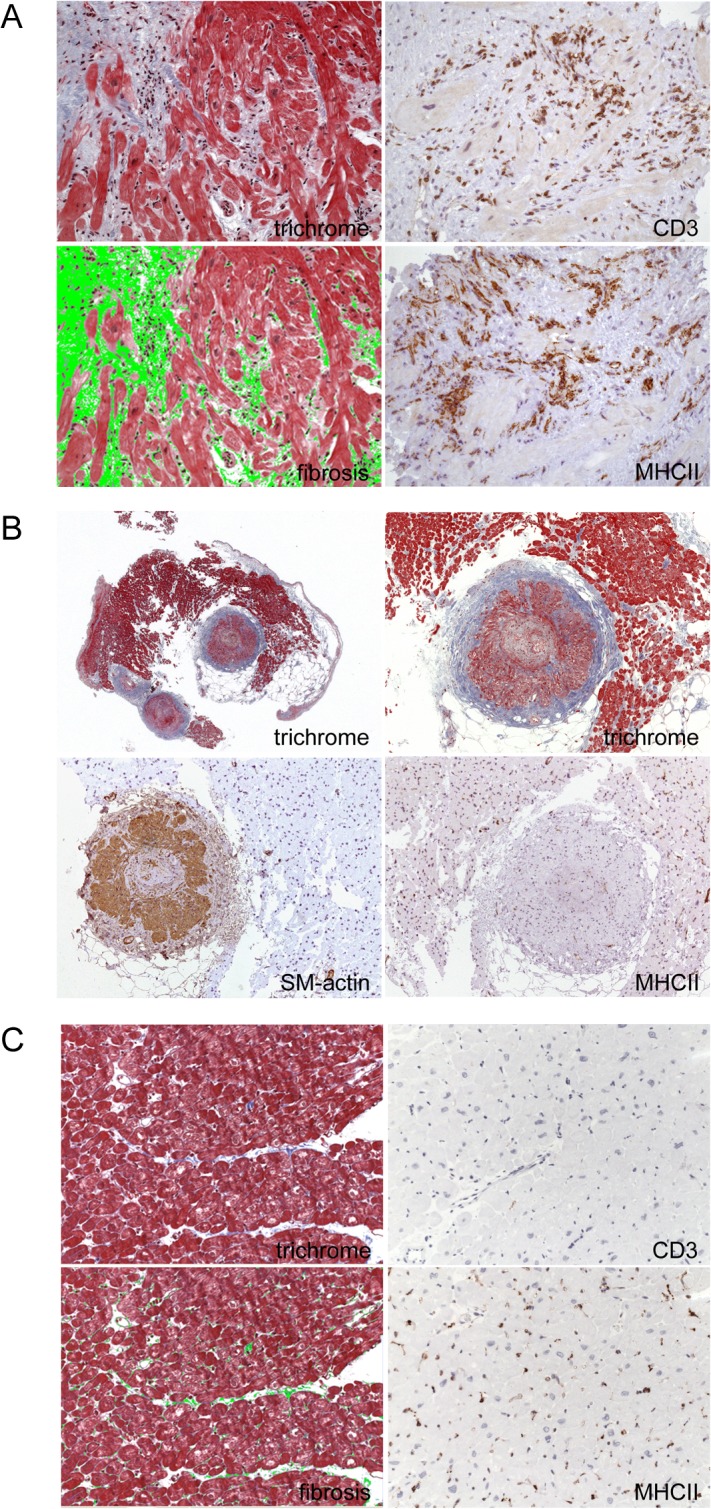
Histopathological and immunohistological findings in the myocardium of patients with systemic sclerosis and cardiac involvement. A: This image is representative for a biopsy with severe inflammation (grade 4), which is characterized by the presence of numerous CD3+ T lymphocytes and MHC II+ macrophages. In addition, a grade 3 fibrosis demonstrates severe cardiac remodeling in this patient. B: In the left picture, an overview of an endomyocardial biopsy is presented revealing two arterioles with pronounced changes of the architecture of the vessel walls including fibrosis. Immunohistological staining with SM-actin confirms a considerable hyperplasia of smooth muscle in the vessel. C: Fig 1C depicts normal heart tissue with Masson’s Trichrome staining on the left side from autopsy material of a patient, who died during an accident. Myocardial fibrosis (green area) was 2%. No inflammation (CD3+ T cells or MHC II+ cells) was detected within the myocardium, as shown in the immunohistochemistry on the right panel.

The amount of cardiac fibrosis was determined by using the interactive imaging analysis system Quantuepatho. Quantuepatho is an interactive computer program generated at the Department of Informatics at the University of Tuebingen, where the cardiopathologist can quantify fibrosis on the basis of Masson’s trichrome (blue) or also sirius red (red) stained tissue. Each tissue section is analyzed by the cardiopathologist, who defines fibrosis on the basis of the fibrous specific staining. Then pictures are taken and the computer program calculates via a chain-code algorithm the fibrous tissue on the basis of the specific blue (in Masson’s trichrome) stained areas, defined directly by the pathologist, and converts it in green areas as demonstrated in Fig 1A. Areas of green (fibrous) tissue are referred to the total area of the tissue section (also determined by the computer). The results are given in area percentage (%) of fibrosis in relation to the total area of the biopsy [37]. According to the amount of fibrosis, patients were categorized using tertile distribution with an equal number of patients in each group. Tertiles were defined as fibrosis grade 1 (0–10%), grade 2 (11–15%) and grade 3 (16–32%).

### Molecular detection of viral genomes

Enteroviruses (comprising coxsackieviruses and echoviruses), adenoviruses, influenza A and B virus, parvovirus B19, human herpes virus type 6, 7 and 8, herpes simplex virus type 1 and 2, human cytomegalovirus, varicella-zoster virus, Epstein-Barr virus were evaluated by nested (RT-) PCR from RNAlater-fixed endomyocardial biopsy samples according to the protocol of the manufacturer (AGS, Heidelberg, Germany) as described [33,38–41]. A biopsy was considered positive for viral infection, if viral genomes were detected by (RT-) PCR and confirmed by sequencing [33,38–41].

### Assessment of Left Ventricular Risk Markers by Contrast-enhanced cardiac magnetic resonance imaging

Cardiac magnetic resonance imaging (CMR) was performed on a 1.5 Tesla (T) scanner (Siemens Medical Systems, Germany) providing a gradient strength of 40 mT/m and maximum slew rate of 200 mT/m/msec. An advanced cardiac software package was used. Images were acquired with the subject in the supine position, by applying electrocardiographically gated breath-hold sequences.

To evaluate functional parameters, the protocol included a breath-hold steady-statefree-precession (SSFP) pulse sequence (repetition time/echo time 3.0/1.5 ms; flip angle 60°, 25 frames per cardiac cycle, matrix 256×192, field of view 300–400 mm) used to acquire cine images in 2-chamber, 4-chamber, short-axis, as well as outflow tract orientation of the right and left ventricle. A stack of contiguous short-axis slices from ventricular apex to base (slice thickness 5 mm, gap 5 mm) was obtained, parallel to the atrioventricular groove, covering the entire left and right ventricle.

Quantitative analysis of functional parameters was performed off-line using dedicated software (ARGUS, Siemens Medical Systems, Germany). End-diastolic volumes (EDV) and end-systolic volumes (ESV) were used to determine left ventricular ejection fraction (LVEF: EDV-ESV/EDV×100). Left ventricular short axis diameter was measured on a midventricular slice position. Left ventricular enddiastolic diameter (LVEDD) was measured using the short-axis slice at the level of the tip of the mitral valve leaflets.

For late gadolinium-enhanced (LGE) imaging a two-dimensional inversion-recovery segmented k-space gradient-echo MR sequence was performed with the following parameters: repetition time/echo time/inversion time 8.0/4.9/240.0–300.0 ms, flip angle 30°, section thickness 8 mm, in-plane resolution 1.2×1.5 mm. For all examinations, the optimal inversion time to suppress the signal of normal myocardium was determined with an inversion recovery prepared SSFP sequence with incrementally increasing inversion times (repetition time/echo time 24/1.12 ms, flip angle 60°, section thickness 8 mm, and inversion times increasing in 20.0 ms increments). CMR images were acquired in short- and long-axis views 10–15 minutes after intravenous injection of 0.15 mmol per kilogram of body weight gadobutrol (Gadovist, Bayer Healthcare, Germany). Total examination time was between 30–45 minutes.

Two experienced investigators independently reviewed the image loops of each subject in a random fashion. For LGE image analysis both readers visually judged the occurrence (presence versus absence), localization, and pattern of LGE. Pattern and extent of LGE were assessed by using short- and long-axis views and were defined as present only if they were detectable in two orthogonal planes. Areas of LGE were allocated to the American Heart Association 17-segment model.

Especially late contrast enhancement is thought to identify areas of myocardial perfusion defects and thus fibrosis and therefore was the major parameter to be evaluated by CMR in addition to left/right ventricular function with ejection fraction and pericardial effusion [42][43][44].

### Statistical analysis

Continuous variables are expressed as mean ± standard deviation and were compared using the student’s t-test. Categorical data are presented as proportions and were analyzed by chi-square test. For this analysis, continuous variables were dichotomized using the patients`median as cut-off values. To evaluate correlations of non-parametric groups we used Crosstabs and Chi-square tests. Statistical analyses were performed using SPSS software version 19.0 (SPSS Inc., Chicago, IL, USA).

## Results

### Patient population, clinical risk parameters and biomarkers

We retrospectively studied a cohort of 25 patients with dc (n = 18) or lc (n = 7) progressive SSc and suspected myocardial involvement. Demographic details and basic characteristics are presented in Table 1 and are given for each single patient in S1 Table.

**Table 1:** Patients’ demographics, treatment, cardiac and inflammatory markers.

*All Patients*	*N = 25*
***Clinical characteristics***	
Mean age, y ± SD	46.0±11.0
Gender, female	8 (32%)
BMI	23.4±4.4
NYHA functional class ≥ II	19 (76%)
NYHA functional class > II	7 (28%)
Subtype (lc/dc)	7 (28%)/18 (72%)
Median disease duration, y	2 (0.5–10)
Median mRSS	16 (3–34)
Renal involvement	2 (8%)
Pulmonary fibrosis (hrCT)	19 (76%)
Oesophageal dysfunction	15 (60%)
Digital ulcers	16 (64%)
Myositis	11 (44%)
Arterial hypertension	5 (20%)
Blood pressure at enrollment	
- Systolic BP (mmHg)	119.6 ± 21.5
- Diastolic BP (mmHg)	71.1 ± 10.4
- MAP (mmHg)	87.3 ± 13.5
Diabetes mellitus type 2	1 (4%)
Hyperlipidemia	5 (20%)
- Triglycerides (mg/dl)	163.0 ± 94.2
- Cholesterol (mg/dl)	198.1 ± 48.3
- HDL (mg/dl)	51.8 ± 18.9
- LDL (mg/dl)	124 ± 31.1
Renal crisis	1 (4%)
***Cardiac medication***	
ß-blockers	14 (56%)
ACE-inhibitors	14 (56%)
AT1-antagonists	5 (20%)
Diuretics	11 (44%)
Aldosterone antagonists	10 (40%)
***Immunosuppressive medication ever***	
Prednisolon	14 (56%)
Cyclophosphamid	24 (96%)
Ciclosporin	2 (8%)
Mycophenolat	15 (60%)
Rituximab	6 (24%)
Methotrexat	5 (20%)
Azathioprin	3 (12%)
***Immunosuppressive medication within the last 6 months before EMB***	
Prednisolon	10 (40%)
Cyclophosphamid	11 (44%)
Ciclosporin	0 (0%)
Mycophenolat	4 (16%)
Rituximab	1 (4%)
Methotrexat	1 (4%)
Azathioprin	0 (0%)
***Medication for pulmonary hypertension***	
Sildenafil	4 (14%)
***Medication for digital ulcers***	
Bosentan	11 (44%)
***Cardiac biomarkers***	
BNP (ng/l)	117.5±111.7
TnI (μg/l)	0.79±1.1
CK (U/l)	407.3±529.7
***Immunological and inflammatory markers***	
ANA positive	20 (80%)
Scl-70 positive	13 (52%)
pmScl positive	1 (4%)
ACA positive	2 (8%)
WBC (n/μl)	10178±2972
ESR (mm/h)	17.0±20.8
CRP (mg/dl)	2.3±3.5

Values are n (%) or mean±standard deviation. ACA – Anti-centromere antibody, ACE inhibitors – angiotensin converting enzyme, ANA – antinuclear antibodies, AT1-antagonists – angiotensin II type 1 receptor antagonist, BMI – body mass index, BNP – b-type natriuretic peptide (normal value < 100ng/l), BP – blood pressure, CRP – C-reactive protein (normal value < 0.5mg/dl), cholesterol—(normal value < 200mg/dl), CK – creatine kinase (normal value < 190U/l), Dc – diffuse cutaneous, dl – deciliters, ESR –erythrocyte sedimentation rate (normal value < 20mm/h), h – hour, HDL – high density lipoprotein (normal value < 40mg/dl), hrCT – high resolution computed tomography, l – liters, lc – limited cutaneous, LDL—low density lipoprotein (normal value < 160mg/dl), mg – milligrams, MAP – mean arterial pressure, mmHg – millimeter of mercury, mRSS – modified Rodnan Skin Score, μg – micrograms, μl – microliters, n – number, ng – nanograms, NYHA – New York Heart Association, pmScl—polymyositis scleroderma antibody, Scl-70 – topoisomerase I, SD – standard deviation, TnI –troponin I (normal value < 0.03µg/l), triglycerides—(normal value < 200mg/dl), U – units, WBC – white blood cells (normal value < 10.000n/μl), y – years. *Continuous variables were compared using t- test, *categorical data were analyzed by chi-square test*.

Out of 25 patients, 8 (32%) were female, mean age at the time of the biopsy was 46.0 ± 11.0 years. The median mRSS was 16 (range from 3 to 34). All patients were tested for ab against PmScl, Anti-Jo and SSA or SSB. ANA – ab were found in 20 (80%) patients. Out of these 20 patients, five (25%) patients showed a homogeneous, seven (35%) a speckled and two (10%) a nucleolar pattern. For the remaining six (30%) patients the ANA pattern was not identified. Only in one patient (4%) we found additional PmScl – ab. Thirteen (52%) patients were Scl-70 positive, while two (8%) patients were found positive for ACA. In 18 (72%) patients CRP (cut-off 0.5 mg/dl) was elevated at the time of biopsy with a mean value of 2.3 ± 3.5 mg/dl. Nineteen (76%) patients in the cohort suffered from pulmonary fibrosis, two (8%) from renal involvement, 16 (64%) from digital ulcers and eleven (44%) showed myositis diagnosed by whole body MRI. In 15 patients (60%) an oesophageal dysfunction was diagnosed by barium-oesophagogram. Five (20%) patients presented with arterial hypertension, which was sufficiently treated at study entry with blood pressure (BP) values < 140/90 mmHg in the documented BP measurements during the hospital stay (Table 1). All patients with arterial hypertension received antihypertensive medication. BP was well controlled at the time of biopsy. There was one (4%) patient with documented diabetes mellitus type 2 in this patient collective and one (4%) patient with renal crisis. Five (20%) patients were diagnosed with hyperlipidemia defined as cholesterol > 200 mg/dl and/or low density lipoprotein (LDL) >160 mg/dl.

Mean BNP was found to be 117.5±111.7 ng/l (cut-off 100ng/l), it was elevated in ten (40%) patients. Mean TnI was increased with 0.79±1.1 μg/l (cut-off 0.03μg/l) in 14 (56%) patients (Table 1).

Couplets and triplets were detected in 39% of the patients in 24h holter recordings. However, ventricular salvos and non sustained ventricular tachycardia (nsVT) were found in 28% of the cohort (Table 2).

**Table 2:** Cardiac work up.

***Echocardiography***	**N = 25**
LVEF (%)	54.1±9.0
LVEDD (mm)	46.7±5.9
RV-function, normal	23 (92%)
RV-function, moderately impaired	1 (4%)
RV-function, severely impaired	1 (4%)
RVEDD (mm)	31.3±5.3
Systolic PAP (mmHg)	36.1±13.1
***Right heart catheterization***	**N = 25**
PAmean (mmHg)	21.1±8.7
***Pulmonary function test***	**N = 25**
FVC (l)	3.1±1.1
FVC (%)	70.8±20.7
***24h-Holter Recording***	**N = 18**
No ventricular ectopy	6 (33%)
Couplets and triplets	7 (39%)
Ventricular salvos or nsVTs	5 (28%)
***Results of endomyocardial biopsies***	
*Degree of inflammation*	N = 25
No inflammation	1 (3.8%)
Single inflammatory cells	10 (38.5%)
Some foci of inflammation	8 (30.8%)
Several foci of inflammation	4 (15.4%)
Pronounced inflammation	2 (7.7%)
*Immunohistology*	N = 25
CD3	14 (56%)
CD68	19 (76%)
MHC II	20 (80%)
*Virus positive*	N = 25
EV	1 (4%)
PVB19	2 (8%)
EBV	2 (8%)
HHV 6	1 (4%)

Values are n (%) or mean±standard deviation. CD – cluster of differentiation, EV – enteroviruses, EBV – Epstein-Barr virus, FVC – functional vital capacity, h – hour, HHV 6 – Human herpesvirus 6, LVEDD – left ventricular enddiastolic diameter, LVEF—left ventricular ejection fraction, MHC II – major histocompatibility complex class II, mm – milimeters, mmHg – milimeters of mercury, nsVTs—non sustained ventricular tachycardia, PAmean—mean pulmonary arterial pressure measured in right heart catheterization, PAP—pulmonary artery pressure,in echocardiography, PVB19—Parvovirus B19, RV—right ventricular, RVEDD – right ventricular enddiastolic diameter, VES – ventricular extrasystoles. *Continuous variables were compared using t- test, *categorical data were analyzed by chi-square test*.

LVEF was slightly impaired with a mean EF of 54.1±9.0% within the patient collective. Only seven patients (28%) showed a moderately to severely reduced LVEF. Reduced RVEF was detected in two patients (8%), a dilated right ventricle (mean RVEDD 31.3±5.3 mm) in 12 patients (48%), while an elevated PAPsys was found in 14 patients (56%), mean PAPsys of 36.1±13.1 mmHg, in echocardiography (Table 2). Right heart catheterization was performed in all patients before endomyocardial biopsy. PAmean was measured in all patients, PAmean is available in 24/25 patients and found to be elevated (PAmean>25mmHg) in five (10%) patients. PAmean was 21.1±8.7 mmHg for the overall cohort.

Pulmonary function tests were performed at baseline in all patients. Mean FVC (l) was 3.1±1.1l, respectively mean FVC (%) 70.7±20.7%.

CMR results were available in 16 (64%) patients, revealing positive LGE in 4/16 (25%) and reduced LVEF in 5/16 (31%) patients, respectively. The median LVEF measured by CMR was 57.5% (range 37–68%). Three out of four patients with positive LGE in CMR showed moderate fibrosis (grade 2) in their histopathological findings. One patient was diagnosed with fibrosis grade 3.

### Histopathological findings of the endomyocardial biopsies

Standard stainings for the histopathologcal evaluation of the endomyocardial biopsies were performed in all 25 patients as described above. The myocardium of all patients revealed fibrotic areas with area percentages ranging from 8% to 32%. In controls of healthy hearts, fibrosis is reported in up to 3% [45].

The degree of inflammation as characterized by the presence of CD3 T-lymphocytes and activated MHC II- positive CD68 macrophages was distributed in the patient collective as follows:

One (3.8%) patient showed no inflammation (grade 0). Single inflammatory cells (grade 1) were found in ten (38.5%) patients. A few foci of inflammation (grade 2) were observed in eight (30.8%) patients, several inflammatory foci (grade 3) in four (15.4%) patients and a pronounced inflammation (grade 4) was detected in two (7.7%) patients (Table 2). The mean grade of inflammation was 1.84. As exemplarily illustrated in Fig 1A inflammatory cells are consistently localized within areas of fibrosis.

Virus genomes were detected in six (24%) patients, but did not correlate with subsequent events during FU (Table 2) corresponding to our previous observations in patients with myocarditis [13]. In the six (24%) patients with virus genome detection we found grade 1 inflammation in four (66.7%) patients and grade 2 inflammation in two (33.3%) patients. The degree of fibrosis was 2 in all four (66.7%) patients with grade 1 inflammation. We detected fibrosis grade 3 in the two (33.3%) remaining patients with grade 2 inflammation.

Fig 1A displays representative images of myocardial tissue of a patient with severe inflammation (grade 4) and grade 3 fibrosis, while Fig 1B demonstrates that also intramyocardial vessels may be involved in disease progression of SSc patients. Immunohistological staining with SM-actin reveals severe changes of the architecture of the vessel wall with a pronounced hyperplasia of smooth muscle cells and fibrosis. For comparison, we provide images from normal heart tissue, illustrating less than 3% fibrosis [45] and absence of inflammation (CD3+ T cells and MHC II expressing macrophages) in Fig 1C.

### Clinical follow-up and cardiovascular events

Patients were seen on a regular schedule for FU in our outpatient clinic after index endomyocardial biopsy, mean FU was 22.5 months.

Cardiovascular events were defined as a combination of cardiovascular death, adequate ICD shock/arrhythmic event, and heart failure-related re-hospitalization.

Due to pathologic findings with evidence of advanced cardiac involvement twelve patients received an ICD for primary prevention of sudden cardiac death.

Seven events occurred (four (16%) cardiovascular death, three (12%) appropriate (ICD-shocks) during FU (Table 3). Six patients died from any cause during FU, four due to cardiovascular causes (three patients were diagnosed with sudden cardiac death by their treating physician, one out of these three patients had documented ventricular tachycardia shortly before death, chest pain, and ecg changes (T-wave inversion in V3—V6, newly diagnosed negative T waves can indicate myocardial ischemia). The fourth patient showed ventricular fibrillation and ventricular tachycardia, which were not adequately terminated by the ICD). One patient suffered from severe dysphagia and died from asphyxia following aspiration of fluids. The last patient died from sepsis during a severe course of pneumonia and showed ventricular tachycardia. There were no hospitalizations due to heart failure or cardiac decompensation during FU.

**Table 3:** Clinical outcome during a mean follow-up of 22.5 months.

*Clinical endpoint*	*N = 25*
Combined endpoint*	7 (28%)
All-cause death	6 (23.1%)
Cardiovascular death	4 (66.7%)
Decompensation due to HF	0 (0%)
ICD-Shock	3 (11.5%)

Values are n (%). HF – heart failure, ICD – implantable cardioverter-defibrillator.

* combination of all-cause death, decompensation due to heart failure and arrhythmic endpoints.

Three (25%) patients with ICD were documented with either ventricular tachycardia or ventricular fibrillation (VT/VF) and were treated with an adequate shock therapy. Two patients were treated with antitachycardia pacing.

**Fig 2:**
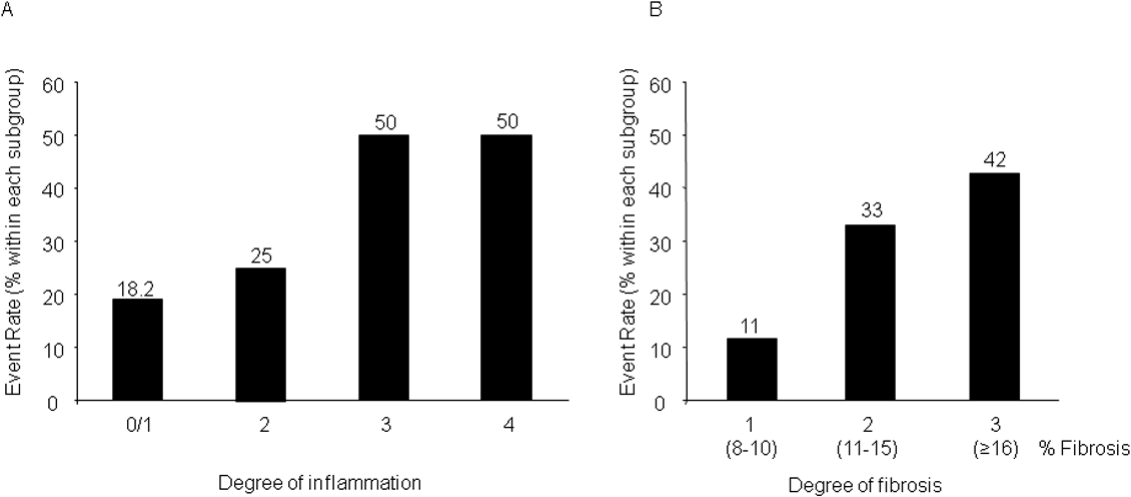
Patients with an event (cardiovascular death, arrhythmic endpoints defined as appropriate discharge of ICD or rehospitalization due to heart failure) during follow up showed a higher grade of inflammation and a higher degree of fibrosis. A: The event rate was associated with the degree of inflammation by trend. While patients with an inflammation grade 0 or 1 showed an event rate of 18.2%, the subgroup of patients with an inflammation grade 2 presented with an event rate of 25% versus an event rate of 50% in the subgroup of patients with an inflammation grade 3 and 4, respectively (p = 0.193). B: The event rate was associated with the degree of fibrosis by trend. While patients with fibrosis grade 1 showed an event rate of 11%, the subgroup of patients with fibrosis grade 2 and 3 presented with an event rate of 33% and 42% respectively (p = 0.160).

Interestingly, the occurrence of any of these events during FU was associated with the degree of myocardial inflammation and fibrosis by trend (Fig 2A and 2B).

While patients with an inflammation grade 0 or 1 showed an event rate of 18.2%, the subgroup of patients with an inflammation grade 2 presented with an event rate of 25% versus an event rate of 50% in the subgroup of patients with an inflammation grade 3 and 4, respectively (p = 0.193). A similar association by trend was observed regarding the degree of myocardial fibrosis. The subgroup of patients with fibrosis grade 1 showed an event rate of 11%, on the other hand patients with fibrosis grade 2 and 3 presented with an event rate of 33% and 42% respectively (p = 0.160).

## Discussion

The principal findings of this observational study are as follows:

First, patients with SSc and suspected cardiac involvement revealed diagnostic findings of impaired LVEF and elevated cardiac biomarkers. Clinical symptoms of heart failure or arrhythmias have been observed in 15% to 35% of patients with SSc [7,8] indicating the importance of clinical screening and diagnostic work-up to detect frequent cardiac disorders in this relevant patient group.

Second, patients with SSc and myocardial involvement have a poor prognosis as documented by an event rate of 28% within 22.5 months follow-up. These observations are in line with previous data documenting up to 70% mortality in SSc with cardiac manifestations [6]. Cardiac involvement in SSc is diverse [46] and can be classified into direct myocardial involvement and the indirect effects caused by other organ involvement (e.g. pulmonary hypertension or renal crisis). Direct cardiac involvement goes along with progressive myocardial fibrosis identified in autopsy studies [47]. Previous data suggest, that signs of myocardial fibrosis are common among patients with SSc. Several former studies including autopsied patients with SSc revealed a prevalence of up to 81% of myocardial fibrosis [47,48]. In our patients we also found a very high prevalence (100%) of cardiac fibrosis, which might be considered as consequence of the observed inflammation in the myocardium. Bulkley et al. described myocardial contraction band necrosis and fibrotic remodeling in 52 autopsied patients [49], while Murata et al. found left ventricular hypertrophy due to endomyocardial, patchy fibrosis [46].

The exact pathogenesis of fibrosis remains uncertain [50]. Early microvascular injury like vasospasm of the small coronary vessels may be the initial event, that leads to ischemia and an activation of the endothelium preceding all pathological changes. Autoimmune and inflammatory responses to the cell damage lead finally to fibroblast activation and their differentiation to myofibroblasts. They are the main source of extracellular matrix protein production leading to myocardial fibrosis [51]. The role of microvascular remodelling is supported by our findings (Fig 1B) and by autopsy findings of concentric intimal hypertrophy in arterioles, while coronary artery disease is absent [52]. But all these observations do not explain entirely the cardiac remodelling due to collagen deposition.

On the other hand, myocardial inflammation has been described in early stages of the disease in various studies [53–55]. Pieroni [56] et al. also showed recently, that in SSc with newly developed symptoms of heart failure and cardiac involvement myocarditis is a common finding. Out of a cohort of 181 SSc patients, they analysed 7 SSc patients with cardiac involvement and examined the efficacy of immunosuppressive therapy. Histopathology revealed upregulation of endothelium adhesion molecules and infiltration of activated T lymphocytes with signs of acute myocarditis in six patients and chronic myocarditis in one patient. Immunosuppressive therapy improved symptoms. In this patient cohort two patients died of sudden death during a follow-up of 5 years. The findings of the small case series of Pieroni et al. support the findings of our present study emphasizing again the poor prognosis of SSc patients with cardiac involvement and the prognostic impact of endomyocardial biopsy.

Various non-invasive methods were evaluated to assess cardiac involvement in Ssc, but none of the performed examinations in our cohort was as sensitive as histopathological analysis. For example, four patients with moderate myocardial fibrosis (10–22% of the myocardium) in histopathology showed only mild clinical symptoms at study entry and only few abnormalities in other examinations. Therefore, performing endomyocardial biopsy is of great use in assuring the diagnosis of cardiac involvement in patients with SSc.

However, the detection rate of cardiac involvement using a single method was poor. Using TTE systolic and diastolic dysfunction, pericardial effusion as well as pulmonary arterial pressure can be assessed. Nevertheless, missing information on conduction disorders or perfusion of the myocardium eludes this method. Thus, we suggest to perform an extensive clinical check up in patients with suspected cardiac involvement including TTE, heart catheterization and endomyocardial biopsy, repeated 24 hour holter ecg, and pulmonary function tests.

Of note, patients with an event in the further course of the disease showed a higher degree of fibrosis and inflammation in their endomyocardial biopsies. Therefore, the histopathological findings may also reveal a prognostic impact in future studies for this patient collective.

Six (24%) patients’ samples had a positive PCR for viral genome. The presence of viral DNA alone (e.g., in persistent virus infection) does not necessarily implicate a cardiac disease [57]. This finding was confirmed by Kindermann et al. 2008 [35], who showed that only the presence of myocardial inflammation allows risk stratification. In contrast, viral genome detection in the myocardium alone was not associated with poor clinical outcome. Also, the frequency of inflammation was similar in virus-positive and virus-negative patients in this cohort. Since we did not observe acute virus infections in our six (24%) patients, but only persistent or latent virus infections, it is very unlikely that inflammatory processes and thus the resulting fibrosis results from the presence of viral DNA in the myocardium.

Another aspect of our findings is, that male SSc patients present significantly more often with severe courses of the disease and therefore, might show more often early cardiac involvement. This explains, why more male patients are included in this study, also regarding further planned aSCT, even though SSc is a disease mainly affecting women. Our results are supported by the findings in the EUSTAR study, the largest worldwide database of SSc patients, that demonstrated a higher risk of severe cardiovascular involvement in men, and raised the suggestion to include sex in the management and the decision-making process in the treatment of SSc patients [58].

Furthermore, we detected 20 patients to be either positive for ANA and/or anti-Scl-70 ab using a cut-off titer of 1:160. Therefore, we report 20% of the patients to be negative for ANAs in the screening of our cohort, an usual finding, as the usual percentage of ANA negativity in most cohorts is not higher than 10%. Recent findings revealed, that ANA negative SSc patients are more commonly male, hence with similar mortality rates as ANA positive patients. ANA negative patients experience less vasculopathic manifestations and PAH was less common, but malabsorption was increased. There was no difference in the frequency of pulmonary fibrosis or renal crisis. All-cause mortality was not different [59]. Cardiac involvement has not been studied specifically in ANA negative SSc patients yet. At this point it is not clear, why ANA positive and ANA negative SSc patients have a similar outcome, cardiac involvement in both cases could be a potential explanation. Since our study is a retrospective analysis, we can not rule out that ANA status might have changed during the course of the disease. Therefore, the high rate of ANA negativity with up to 20% should not be overinterpreted.

Due to its retrospective character and the small sample size as well as the missing control group, the significance of our study is limited. In addition, our cohort represents a negative selection as we considered especially patients with severe Ssc and poor prognosis. Another limitation is that patients, who died during follow up, did not undergo autopsy post mortem. Therefore, there is no information available to correlate the degree of subendocardial fibrosis post mortem on a global scale with the degree of fibrosis found in our endomyocardial biopsies, even though it would be of great interest to evaluate autopsy slides from this patient collective. Nevertheless, this is the first study on endomyocardial biopsy in patients with severe SSc and the findings are impressive as cardiac fibrosis was found in 100% and we show a noticeable relationship of the degree of inflammation and fibrosis with the composite primary endpoint.

Of course, there are also limitations regarding the impact of the endomyocardial biopsy. According to the recent Association for European Cardiovascular Pathology guidelines, for the histological, immunohistochemical, and molecular diagnosis of inflammatory myocardial disease, optimal specimen procurement and triage indicates at least three fragments gained in endomyocardial biopsy, each 1–2 mm in size, for light microscopic examination [31,32].Since there is the possibility of focal inflammation and fibrosis in the heart of patients, it is likely, that some positive findings regarding inflammation and fibrosis are not detected in the biopsies investigated. With regard to the findings in our study this would mean, that in some cases inflammation and fibrosis is underestimated. However, as we have already significant results regarding inflammation and fibrosis in our SSc patients compared to control patients, an underestimation would not downgrade the present findings.

A larger prospective cohort study is needed to evaluate additional independent predictors for clinical outcome in patients with SSc and cardiac involvement. However, the present study suggests, that endomyocardial biopsy complements the risk assessment of clinical presentation, imaging and functional parameters as an additional tool to identify high risk patients at an early stage of the disease or in preparation of aSCT to minimize cardiac complications. Additional parameters to delineate pathogenetic mechanisms of myocardial fibrosis and inflammation might be of further benefit to specify cardiac risk, in particular sudden cardiac death [10,11,13,60,61]. Combining the results of non-invasive imaging modalities, histopathologic findings and electrophysiologic characterization may further improve risk stratification for sudden cardiac death in this patient population [23]. This strategy would help to develop algorithms to identify risk patients with SSc, who might benefit from early primary prophylactic ICD implantation, independent of current guideline recommendation using left ventricular ejection fraction as main criterion for patient selection.

We are aware, that we might have missed relevant arrhythmic endpoints in our patient cohort, as not all patients were monitored by cardiac devices. But the majority of patients (n = 18, 72%) was monitored with repeated holter ecg during the follow up of the study.

We do not recommend endomyocardial biopsy in all Ssc patients in clinical routine, but an early detection of cardiac involvement may be fundamental to improve mortality rate, especially prior to an aggressive therapeutic approach such as aSCT or even a cyclophosphamide (CYC) pulse therapy.

ASCT has been shown to be a very feasible and effective treatment option for selected patients with severe SSc [11,62,63]. Cardiac involvement is associated with increased mortality, in fact, it is one of the leading causes of death in patients with SSc [2,4,64], and can be a risk factor for cardiac deaths even after successful aSCT [11]. Therefore, an extensive workup, including CMR, TTE, and right and left heart catheterization has been suggested before considering aSCT [62,65]. Regarding the findings in our study, we also advocate to perform endomoycardial biopsy before aSCT to intensify the risk assessment and for a thorough evaluation for primary cardiac disease and/or constrictive pericarditis, coronary artery disease, hypertensive cardiomyopathy, or PAH [62]. Ssc patients can show various cardiac manifestations, therefore it is of great importance to evaluate the origin of cardiac manifestations or even co-morbidities [50].

Regarding findings of previous intervention trials in Ssc, one has to keep in mind, that CYC is one cornerstone in the treatment of SSc and is used in most protocols for aSCT in SSc [2,11,62,63,66,67,68], despite its known cardiotoxic effects [69–71]. The toxicity profile of CYC is typically acute and dose dependent, life-threatening cardiotoxicity of CYC has been described at the high dosage [71,72]. Another aspect is, that before being considered for aSCT, most patients have already been exposed to CYC as standard treatment [71,72]. It is presumed that the cytotoxic effect of CYC is proportional to the area under the curve (AUC) values of the active metabolite 4-hydroxycyclophosphamide/aldo- phosphamide [72]. Therefore the especially high-dose therapy with CYC used for conditioning in aSCT with 4x50 mg/kg body weight represents a risk factor, particularly in SSc patients with pre-existing myocardial manifestations. Cumulative toxicity is minimal. Moreover, a correlation between peak concentration and cardiac toxicity has been reported, possibly indicating a role of infusion rate in side effects [72]. A recent study could show, that a therapy regimen with adding thiotepa, an alkylating agent, that is used in haematological malignancies for aSCT and has no known cardiotoxicity, could be used successfully in patients with SSc and cardiac involvement [73]. The time to haematological recovery was was longer compared to CYC treatment and infectious complications were described. Hence, there was no transplant-related mortality observed. During a median follow-up of 1.6 years the six patients included in the study did not show an elevation of cardiac biomarkers or a reduction of left ventricular function. For safety precautions, primary prevention ICDs were implanted in all patients before aSCT. Three of six patients (50%) experienced an adequate ICD therapy due to VTs during or after aSCT. This high ICD intervention rate (50%) underlines the risk of conduction irregularities in SSc patients with cardiac manifestations. As a consequence, we advocate an early ICD implantation in those patients before aSCT, suggesting that ICD implantation is even more essential than the reduction of CYC, as Rosen et al. [74] could not find evidence of cardiotoxicity in the autopsy on a patient who died after aSCT.

Cardiac assessment is therefore inevitable, in particular in those patients pre-treated with CYC, given both the frequent and often subclinical cardiac involvement with a pleomorphic picture ranging from microvascular injury to patchy fibrosis involving both the myocardium and the conducting system in this disease [75] and the known acute cardiotoxicity of CYC as a number of clinical reports of severe/fatal toxicity described [67,69,71,76–79]. The results from the randomized clinical trials showed, that patient selection is a critical point to achieve favourable outcome in transplanted SSc patients, in which additional risks are described [62,65,80,81].

In conclusion, this study is a hypothesis-generating observational study to stimulate further research for individual risk assessment in this poorly understood and jeopardized group of patients with SSc and cardiac involvement. To date, there are only single case reports and small case series, that did not systemically evaluate the presence and prognostic impact of histological and clinical risk parameters. Therefore, although still limited in number, the present analysis represents the largest consecutive and systematic assessment of histological findings and clinical risk factors and their association with clinical endpoints. On the basis of these findings, larger registries have to focus on the role of standardized risk assessment including evaluation of inflammatory and fibrotic degree in cardiac histology. Thus, early risk assessment might lead to early and individually targeted therapy in SSc patients to improve prognosis. In particular, the proposed diagnostic characteristics in endomyocardial biopsy might help to detect cardiac involvement at an early stage of the disease.

## Supporting Information

S1 Table Baseline characteristics and clinical parameters of each patient in the cohort. Values are n (%) or mean±standard deviation. ANA – antinuclear antibodies, AVB – AV block, BNP – brain natriuretic peptide, bpm – beats per minute, CK – creatine kinase, Dc – diffuse cutaneous, Dur. – duration of disease, ECG – electrocardiogram, FVC – functional vital capacity, HR – heart rate per minute, hrCT – high resolution computed tomography, lc – limited cutaneous, LAFB – Left Anterior Fascicular Block, LBBB- Left Bundle Branch Block, LGE – late gadolinium enhancement, LVF – left ventricular ejection fraction, LVEDD—left ventricular end diastolic diameter, mRSS – modified Rodnan Skin Score, CMR – cardiac magnetic resonance imaging, neg.—negative, NYHA – New York Heart Association, PAmean – mean pulmonary arterial pressure measured in right heart catheterization, PF – pulmonary fibrosis diagnosed in hrCT, Pts—patients, RBBB- Right Bundle Branch Block, RVF – right ventricular ejection fraction, RVEDD – right ventricular end diastolic diameter, Scl-70—Topoisomerase, SD – standard deviation, SR – sinusrhythm, Ssc – systemic sclerosis, T – T wave, TnI –troponin I, y – years. *Continuous variables were compared using t- test, categorical data were analyzed by chi-square test.
(DOC)
