# Supplementary material for: Clinical and Histopathological Features of Patients with Systemic Sclerosis Undergoing Endomyocardial Biopsy
Source: PLoS One. 2015 May 12;10(5):e0126707. doi: 10.1371/journal.pone.0126707 (PMC4428754; doi:10.1371/journal.pone.0126707)
Supplement: S1 Table — Values are n (%) or mean±standard deviation. ANA – antinuclear antibodies, AVB – AV block, BNP – brain natriuretic peptide, bpm – beats per minute, CK – creatine kinase, Dc – diffuse cutaneous, Dur. – duration of disease, ECG – electrocardiogram, FVC – functional vital capacity, HR – heart rate per minute, hrCT – high resolution computed tomography, lc – limited cutaneous, LAFB – Left Anterior Fascicular Block, LBBB- Left Bundle Branch Block, LGE – late gadolinium enhancement, LVF – left ventricular ejection fraction, LVEDD—left ventricular end diastolic diameter, mRSS – modified Rodnan Skin Score, CMR – cardiac magnetic resonance imaging, neg.—negative, NYHA – New York Heart Association, PAmean – mean pulmonary arterial pressure measured in right heart catheterization, PF – pulmonary fibrosis diagnosed in hrCT, Pts—patients, RBBB- Right Bundle Branch Block, RVF – right ventricular ejection fraction, RVEDD – right ventricular end diastolic diameter, Scl-70—Topoisomerase, SD – standard deviation, SR – sinusrhythm, Ssc – systemic sclerosis, T – T wave, TnI –troponin I, y – years. *Continuous variables were compared using t- test, categorical data were analyzed by chi-square test. (DOC) [file pone.0126707.s001.doc]

Supplemental Table S1: Baseline characteristics and clinical parameters of each patient in the cohort

| **Pts.** | **Sex** | **Age** | **Dur. Ssc (ys)** | **Ssc lc/dc** | **NYHA** | **ANA**  **+/-** | **Scl70 +/-** | **BNP ng/l** | **TnI µg/l** | **CK U/l** | **PF +/-** | **LVF (%)** | **LVEDD**  **mm** | **PE**  **+/-** | **RVEDD mm** | **RVF**  **+/-** | **CMRLGE** | **Inflammation**  **0-4** | **Fibrosis**  **+/++/+++** | **24h ECG**  **Lown Class** | **PAmean mmHg** | **FVC (l)** | **FVC (%)** | **ECG**  **( time in ms)** |
| --- | --- | --- | --- | --- | --- | --- | --- | --- | --- | --- | --- | --- | --- | --- | --- | --- | --- | --- | --- | --- | --- | --- | --- | --- |
| 1 | f | 53 | 9 | lc | 3 | + | - | 57 | <0.03 | 61 | + | 66 | 41 | - | 29 | + | ND | 1 | + | ND | 21 | 2.56 | 77.7 | SR, 66bpm, PQ 166, QRS 86, |
| 2 | m | 42 | 6 | dc | 1 | - | - | 14 | <0.03 | 574 | + | 53 | 49 | - | 28 | - | - | 2 | ++ | 1 | 14 | .3.21 | 54.6 | SR, 76bpm, PQ 138, QRS 82 |
| 3 | m | 43 | 2 | dc | 2 | + | + | 175 | 0,88 | 668 | + | 40 | 57 | - | 42 | - | ND | 2 | +++ | 4a | 16 | 5.2 | 88.7 | SR, 81bpm, PQ 206, AVB I°, QRS 94 |
| 4 | m | 50 | 8 | dc | 2 | + | + | 63 | <0.03 | 142 | + | 65 | 56 | - | 28 | - | - | 3 | ++ | 4a | 14 | 2.9 | 56 | SR, 73bpm, PQ 136, QRS 104, neg. T in III |
| 5 | m | 38 | 2 | dc | 4 | + | + | 61 | 0,16 | 160 | - | 23 | 55 | - | 27 | - | ND | 2 | +++ | ND | 30 | 4.05 | 104 | SR, 128bpm, PQ 228, AVB I°, QRS 70 |
| 6 | f | 50 | 10 | lc | 3 | + | - | 90 | 3,6 | 709 | - | 55 | 43 | - | 25 | - | - | 1 | ++ | 4b | 18 | 2.24 | 72 | Atrial flutter 2:1conduction, 94bpm, QRS 130, LAFB, RBBB |
| 7 | f | 44 | 1 | dc | 1 | + | + | 21 | <0.03 | 51 | + | 55 | 44 | - | 22 | - | - | 2 | ++ | 4a | 14 | 2.36 | 71.6 | SR, 88bpm, PQ 120, QRS 90, neg T in III, V2 |
| 8 | m | 56 | 1,5 | dc | 1 | + | + | 18 | 0,12 | 304 | + | 55 | 49 | - | 34 | + | - | 1 | +++ | 4a | 40 | 2.54 | 55.6 | SR, 70bpm, PQ 200, AVB I°, QRS 100, LAFB |
| 9 | m | 48 | 3 | dc | 2 | + | + | 232 | 0,18 | 100 | + | 50 | 37 | - | 31 | - | + | 4 | +++ | 4a | 25 | 2.7 | 56.2 | SR, 104bpm, PQ 180, QRS 80, LAFB, R/S V5/V6, neg T inV2-V6 |
| 10 | m | 49 | 2 | dc | 2 | + | - | 50 | <0.03 | 829 | + | 60 | 48 | - | 32 | - | ND | 1 | ++ | 4a | 21 | 3.6 | 77 | SR, 85bpm, PQ 164, QRS 102, LAFB |
| 11 | m | 43 | 9 | dc | 2 | - | - | 41 | <0.03 | 64 | + | 60 | 46 | - | 36 | - | ND | 1 | ++ | ND | 23 | 2.7 | 49.6 | SR, 60bpm, PQ 140, QRS 144, LBBB |
| 12 | m | 23 | 0,5 | dc | 1 | + | + | 82 | 0,48 | 378 | - | 60 | 44 | - | 35 | - | - | 2 | +++ | 4a | 24 | 5.66 | 113 | SR, 86bpm, PQ 144, QRS 84 |
| 13 | f | 19 | 7 | dc | 3 | + | + | 12 | 0,2 | 109 | + | 55 | 40 | - | 20 | - | - | 2 | ++ | 1 | 18 | 1.92 | 51 | SR, 78bpm, PQ 114, QRS106 |
| 14 | m | 46 | 7 | lc | 2 | + | - | 166 | 0,11 | 91 | - | 55 | 55 | - | 35 | - | ND | 1 | ++ | ND | 17 | 4.73 | 89.3 | SR, 64bpm, PQ 260, AVB I°, QRS 168, LBBB |
| 15 | f | 49 | 9 | dc | 3 | + | + | 126 | <0.03 | 53 | + | 40 | 48 | - | 30 | - | - | 3 | +++ | 4b | 19 | 1.46 | 46.9 | SR, 78bpm, PQ 190, QRS 130, LBBB |
| 16 | m | 49 | 3 | dc | 2 | + | + | 17 | <0.03 | 30 | - | 60 | 50 | - | 39 | - | ND | 1 | ++ | 1 | 24 | 5.66 | 113 | SR, 72bpm, PQ 144, QRS 90 |
| 17 | m | 47 | 1 | lc | 1 | + | - | 355 | <0.03 | 37 | - | 55 | 36 | + | 35 | - | - | 1 | ++ | 1 | 50 | 3.16 | 58.8 | Atrial fibrillation, 90bpm, QRS 95 |
| 18 | f | 68 | 1 | lc | 1 | + | - | 137 | 0,21 | 846 | + | 55 | 46 | - | 36 | - | ND | 3 | ++ | ND | 30 | 3.21 | 102.8 | SR, 60bpm, PQ190, QRS 100 |
| 19 | m | 36 | 1 | dc | 2 | + | + | 171 | 0,66 | 323 | + | 50 | 44 | - | 30 | - | - | 3 | +++ | 4b | 12 | 3.02 | 66.4 | SR, 68bpm, PQ 210, AVB I°, QRS 110, LAFB |
| 20 | m | 54 | 2,5 | lc | 3 | + | - | 4 | <0.03 | 78 | + | 60 | 42 | - | 30 | - | + | 1 | ++ | ND | 16 | 3.94 | 87.9 | SR, 85bpm, PQ 136, QRS 126, RBBB |
| 21 | f | 43 | 1 | dc | 2 | - | - | 363 | 1,61 | 1103 | + | 50 | 40 | + | 29 | - | + | 1 | ++ | 4b | 15 | 1.19 | 32.6 | SR, 131bpm, PQ 136, QRS 76 |
| 22 | m | 57 | 0,5 | dc | 3 | + | + | 315 | 1,17 | 2520 | + | 55 | ND | + | 40 | - | + | 2 | ++ | 4b | 12 | 1.94 | 44.6 | SR, 76bpm, PQ 172, QRS 92, neg. T in III |
| 23 | f | 41 | 2 | lc | 2 | - | - | 13 | <0.03 | 30 | + | 60 | 48 | - | 32 | - | - | 0 | + | 1 | 18 | 2.96 | 84 | SR, 74bpm, PQ 130, QRS 80 |
| 24 | m | 65 | 1 | dc | 2 | - | - | 304 | 0,15 | 355 | + | 60 | 51 | - | 28 | - | ND | 2 | ++ | ND | 20 | 2.81 | 64.7 | SR, 60bpm, PQ 180, QRS 70 |
| 25 | m | 39 | 1 | dc | 2 | + | + | 79 | 0,22 | 344 | + | 55 | 52 | - | 30 | - | - | 4 | ++ | 0 | 20 | 4.85 | 91.5 | SR, 60bpm, PQ 220, AVB I°, QRS 120, LBBB |

Values are n (%) or mean±standard deviation. ANA – antinuclear antibodies, AVB – AV block, BNP – brain natriuretic peptide, bpm – beats per minute, CK – creatine kinase, Dc – diffuse cutaneous, Dur. – duration of disease, ECG – electrocardiogram, FVC – functional vital capacity, HR – heart rate per minute, hrCT – high resolution computed tomography, lc – limited cutaneous, LAFB – Left Anterior Fascicular Block, LBBB- Left Bundle Branch Block, LGE – late gadolinium enhancement, LVF – left ventricular ejection fraction, LVEDD - left ventricular end diastolic diameter, mRSS – modified Rodnan Skin Score, CMR – cardiac magnetic resonance imaging, neg. - negative, NYHA – New York Heart Association, PAmean – mean pulmonary arterial pressure measured in right heart catheterization, PF – pulmonary fibrosis diagnosed in hrCT, Pts - patients, RBBB- Right Bundle Branch Block, RVF – right ventricular ejection fraction, RVEDD – right ventricular end diastolic diameter, Scl-70 - Topoisomerase, SD – standard deviation, SR – sinusrhythm, Ssc – systemic sclerosis, T – T wave, TnI –troponin I, y – years.

**Continuous variables were compared using t- test, categorical data were analyzed by chi-square test.*
